# 4-Chloro-*N*-(3-methyl­benzo­yl)benzene­sulfonamide monohydrate

**DOI:** 10.1107/S1600536811051932

**Published:** 2011-12-07

**Authors:** P. A. Suchetan, Sabine Foro, B. Thimme Gowda, M. Shet Prakash

**Affiliations:** a Department of Chemistry, Mangalore University, Mangalagangotri 574 199, Mangalore, India; b Institute of Materials Science, Darmstadt University of Technology, Petersenstrasse 23, D-64287 Darmstadt, Germany; c Department of Chemistry, University College of Science, Tumkur University, Tumkur 572 102, India

## Abstract

In the title compound, C_14_H_12_ClNO_3_S·H_2_O, the dihedral angle between the sulfonyl and benzoyl benzene rings is 84.4 (2)°. In the crystal, every water mol­ecule forms four hydrogen bonds with three different mol­ecules of 4-chloro-*N*-(3-methyl­benzo­yl)benzene­sulfonamide. One of the water H atoms forms a bifurcated hydrogen bond with both the sulfonyl and the carbonyl O atoms of the same mol­ecule. Mol­ecules are linked into layers in the *ab* plane through N—H⋯O and O—H⋯O hydrogen bonds.

## Related literature

For our studies on the effects of substituents on the structures and other aspects of *N*-(ar­yl)-amides, see: Gowda *et al.* (2004 ▶), on *N*-(ar­yl)-methane­sulfonamides, see: Jayalakshmi & Gowda (2004 ▶), on *N*-(ar­yl)-aryl­sulfonamides, see: Gowda *et al.* (2003 ▶), on *N*-(substitutedbenzo­yl)-aryl­sulfonamides, see: Suchetan *et al.* (2011 ▶) and on *N*-chloro­aryl­amides, see: Gowda *et al.* (1996 ▶).
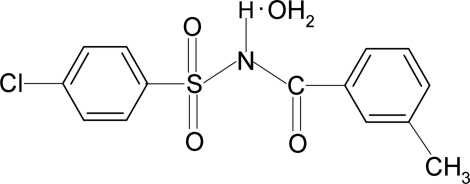


## Experimental

### 

#### Crystal data

C_14_H_12_ClNO_3_S·H_2_O
*M* *_r_* = 327.77
Orthorhombic, 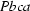

*a* = 5.0148 (6) Å
*b* = 12.864 (2) Å
*c* = 46.314 (5) Å
*V* = 2987.7 (7) Å^3^
*Z* = 8
Mo *K*α radiation
μ = 0.41 mm^−1^
*T* = 293 K
0.46 × 0.14 × 0.06 mm

#### Data collection

Oxford Diffraction Xcalibur diffractometer with a Sapphire CCD detector
Absorption correction: multi-scan (*CrysAlis RED*; Oxford Diffraction, 2009 ▶) *T*_min_ = 0.834, *T*_max_ = 0.976
5972 measured reflections
2684 independent reflections
1959 reflections with *I* > 2σ(*I*)
*R*_int_ = 0.037

#### Refinement

*R*[*F* ^2^ > 2σ(*F* ^2^)] = 0.105
*wR*(*F* ^2^) = 0.185
*S* = 1.35
2684 reflections
200 parameters
3 restraints
H atoms treated by a mixture of independent and constrained refinement
Δρ_max_ = 0.33 e Å^−3^
Δρ_min_ = −0.34 e Å^−3^

### 

Data collection: *CrysAlis CCD* (Oxford Diffraction, 2009 ▶); cell refinement: *CrysAlis RED* (Oxford Diffraction, 2009 ▶); data reduction: *CrysAlis RED*; program(s) used to solve structure: *SHELXS97* (Sheldrick, 2008 ▶); program(s) used to refine structure: *SHELXL97* (Sheldrick, 2008 ▶); molecular graphics: *PLATON* (Spek, 2009 ▶); software used to prepare material for publication: *SHELXL97*.

## Supplementary Material

Crystal structure: contains datablock(s) I, global. DOI: 10.1107/S1600536811051932/bt5739sup1.cif

Structure factors: contains datablock(s) I. DOI: 10.1107/S1600536811051932/bt5739Isup2.hkl

Supplementary material file. DOI: 10.1107/S1600536811051932/bt5739Isup3.cml

Additional supplementary materials:  crystallographic information; 3D view; checkCIF report

## Figures and Tables

**Table 1:** Hydrogen-bond geometry (Å, °)

*D*—H⋯*A*	*D*—H	H⋯*A*	*D*⋯*A*	*D*—H⋯*A*
N1—H1*N*⋯O4	0.86 (2)	1.93 (2)	2.771 (8)	169 (6)
O4—H41⋯O1^i^	0.84 (2)	2.29 (7)	2.916 (7)	131 (8)
O4—H41⋯O3^i^	0.84 (2)	2.42 (6)	3.117 (8)	140 (8)
O4—H42⋯O2^ii^	0.84 (2)	2.35 (6)	3.022 (8)	137 (8)

Symmetry codes: (i) 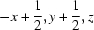; (ii) 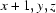.

**Fig. 1.**
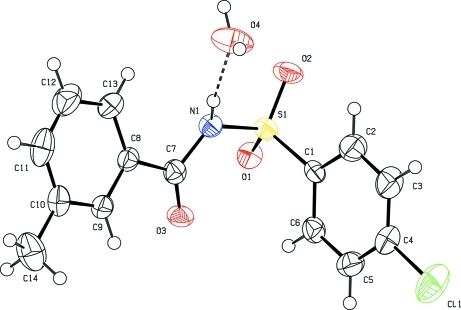
Molecular structure of the title compound, showing the atom-labelling scheme. Displacement ellipsoids are drawn at the 50% probability level.

**Fig. 2.**
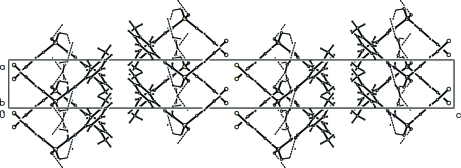
Molecular packing in the title compound. Hydrogen bonds are shown as dashed lines.

## supplementary crystallographic information

### Comment

Diaryl acylsulfonamides are known as potent antitumor agents. As part of our studies on the substituent effects on the structures and other aspects of *N*-(aryl)-amides (Gowda *et al.*, 2004), *N*-(aryl)-methanesulfonamides (Jayalakshmi & Gowda, 2004), *N*-(aryl)-arylsulfonamides (Gowda *et al.*, 2003); *N*-(substitutedbenzoyl)-arylsulfonamides (Suchetan *et al.*, 2011) and *N*-chloro-arylsulfonamides (Gowda *et al.*, 1996), in the present work, the crystal structure of 4-Chloro-*N*-(3-methylbenzoyl)-benzenesulfonamide monohydrate (I) has been determined (Fig.1).

The conformations of the N—H and C=O bonds in the C—SO_2_—NH—C(O) segment are *anti* to each other (Fig.1), similar to that observed in 4-Chloro-*N*-(2-methylbenzoyl)-benzenesulfonamide monohydrate (II) (Suchetan *et al.*, 2011). The molecule is twisted at the *S*- atom with the torsional angle of -70.67 (55)°, compared to the value of -69.2 (2)° in (II).

The dihedral angle between the sulfonyl benzene ring and the —SO_2_—NH—C—O segment is 78.4 (2)°, compared to the value of 87.2 (1)° in (II). Furthermore, the dihedral angle between the sulfonyl and the benzoyl benzene rings is 84.4 (2)°, compared to the value of 57.7 (1)° in (II).

Further, the crystal structure shows interesting H-bonding. Every water molecule forms four H-bonds with three different molecules of the title compound. One of the H-atoms of the water molecule forms simultaneous H-bonding with both the sulfonyl and the carbonyl oxygen atoms of the same molecule.

The packing of molecules through N1—H1N···O4, O4—H41···O1, O4—H41···O3 and O4—H42···O2 hydrogen bonds (Table 1) is shown in Fig. 2.

### Experimental

The title compound was prepared by refluxing a mixture of *m*-methyl benzoic acid (0.02 mole), 4-chlorobenzenesulfonamide (0.02 mole) and excess phosphorous oxy chloride for 3 h on a water bath. The resultant mixture was cooled and poured into crushed ice. The solid, 4-Chloro-*N*-(3-methylbenzoyl)-benzenesulfonamide monohydrate, obtained was filtered, washed thoroughly with water and then dissolved in sodium bicarbonate solution. The compound was later reprecipitated by acidifying the filtered solution with dilute HCl. It was filtered, dried and recrystallized.

Needle like colourless single crystals of the title compound used in X-ray diffraction studies were obtained by slow evaporation of its ethanol-tetrahydrofuran solution at room temperature.

### Refinement

The H atoms of the NH group and of the water molecule were located in a difference map and later restrained to N—H = 0.86 (2)Å and O—H = 0.85 (2) Å. The other H atoms were positioned with idealized geometry using a riding model with the aromatic C—H = 0.93 Å and methyl C—H = 0.96 Å. All H atoms were refined with isotropic displacement parameters set to 1.2 times of the *U*_eq_ of the parent atom.

### Crystal data

**Table:**

C_14_H_12_ClNO_3_S·H_2_O	*F*(000) = 1360
*M**_r_* = 327.77	*D*_x_ = 1.457 Mg m^−^^3^
Orthorhombic, *P**b**c**a*	Mo *K*α radiation, λ = 0.71073 Å
Hall symbol: -P 2ac 2ab	Cell parameters from 1913 reflections
*a* = 5.0148 (6) Å	θ = 2.6–27.8°
*b* = 12.864 (2) Å	µ = 0.41 mm^−^^1^
*c* = 46.314 (5) Å	*T* = 293 K
*V* = 2987.7 (7) Å^3^	Needle, colourless
*Z* = 8	0.46 × 0.14 × 0.06 mm

### Data collection

**Table:**

Oxford Diffraction Xcalibur diffractometer with a Sapphire CCD detector	2684 independent reflections
Radiation source: fine-focus sealed tube	1959 reflections with *I* > 2σ(*I*)
graphite	*R*_int_ = 0.037
Rotation method data acquisition using ω and phi scans	θ_max_ = 25.4°, θ_min_ = 2.6°
Absorption correction: multi-scan (*CrysAlis RED*; Oxford Diffraction, 2009)	*h* = −3→6
*T*_min_ = 0.834, *T*_max_ = 0.976	*k* = −9→15
5972 measured reflections	*l* = −55→55

### Refinement

**Table:**

Refinement on *F*^2^	Primary atom site location: structure-invariant direct methods
Least-squares matrix: full	Secondary atom site location: difference Fourier map
*R*[*F*^2^ > 2σ(*F*^2^)] = 0.105	Hydrogen site location: inferred from neighbouring sites
*wR*(*F*^2^) = 0.185	H atoms treated by a mixture of independent and constrained refinement
*S* = 1.35	*w* = 1/[σ^2^(*F*_o_^2^) + (0.*P*)^2^ + 17.7362*P*] where *P* = (*F*_o_^2^ + 2*F*_c_^2^)/3
2684 reflections	(Δ/σ)_max_ = 0.001
200 parameters	Δρ_max_ = 0.33 e Å^−^^3^
3 restraints	Δρ_min_ = −0.34 e Å^−^^3^

### Special details

**Table:**

Experimental. CrysAlis RED (Oxford Diffraction, 2009) Empirical absorption correction using spherical harmonics, implemented in SCALE3 ABSPACK scaling algorithm.
Geometry. All e.s.d.'s (except the e.s.d. in the dihedral angle between two l.s. planes) are estimated using the full covariance matrix. The cell e.s.d.'s are taken into account individually in the estimation of e.s.d.'s in distances, angles and torsion angles; correlations between e.s.d.'s in cell parameters are only used when they are defined by crystal symmetry. An approximate (isotropic) treatment of cell e.s.d.'s is used for estimating e.s.d.'s involving l.s. planes.
Refinement. Refinement of *F*^2^ against ALL reflections. The weighted *R*-factor *wR* and goodness of fit *S* are based on *F*^2^, conventional *R*-factors *R* are based on *F*, with *F* set to zero for negative *F*^2^. The threshold expression of *F*^2^ > σ(*F*^2^) is used only for calculating *R*-factors(gt) *etc*. and is not relevant to the choice of reflections for refinement. *R*-factors based on *F*^2^ are statistically about twice as large as those based on *F*, and *R*- factors based on ALL data will be even larger.

### Fractional atomic coordinates and isotropic or equivalent isotropic displacement parameters (Å^2^)

**Table:**

	*x*	*y*	*z*	*U*_iso_*/*U*_eq_	
C1	0.3419 (12)	−0.0253 (5)	0.07777 (12)	0.0283 (14)	
C2	0.4759 (15)	0.0400 (6)	0.05920 (14)	0.0469 (19)	
H2	0.4533	0.1117	0.0604	0.056*	
C3	0.6448 (16)	−0.0027 (6)	0.03877 (15)	0.051 (2)	
H3	0.7378	0.0403	0.0262	0.061*	
C4	0.6751 (15)	−0.1085 (6)	0.03706 (14)	0.0423 (18)	
C5	0.5420 (15)	−0.1736 (6)	0.05504 (14)	0.0437 (18)	
H5	0.5650	−0.2451	0.0536	0.052*	
C6	0.3717 (14)	−0.1325 (5)	0.07558 (13)	0.0367 (16)	
H6	0.2776	−0.1764	0.0879	0.044*	
C7	0.4414 (13)	−0.0406 (5)	0.14890 (13)	0.0323 (15)	
C8	0.6298 (13)	−0.0066 (5)	0.17281 (13)	0.0308 (15)	
C9	0.7972 (13)	−0.0844 (5)	0.18361 (13)	0.0335 (16)	
H9	0.7884	−0.1511	0.1759	0.040*	
C10	0.9761 (14)	−0.0630 (6)	0.20566 (14)	0.0403 (18)	
C11	0.9834 (16)	0.0360 (7)	0.21646 (15)	0.055 (2)	
H11	1.1054	0.0517	0.2309	0.066*	
C12	0.8156 (18)	0.1131 (7)	0.20656 (16)	0.059 (2)	
H12	0.8217	0.1791	0.2148	0.071*	
C13	0.6372 (16)	0.0924 (5)	0.18426 (14)	0.0437 (18)	
H13	0.5253	0.1441	0.1772	0.052*	
C14	1.1601 (16)	−0.1458 (7)	0.21706 (17)	0.063 (2)	
H14A	1.2381	−0.1830	0.2012	0.075*	
H14B	1.0610	−0.1933	0.2289	0.075*	
H14C	1.2986	−0.1141	0.2283	0.075*	
N1	0.3383 (10)	0.0427 (4)	0.13292 (11)	0.0302 (12)	
H1N	0.429 (11)	0.099 (3)	0.1325 (14)	0.036*	
O1	−0.0632 (9)	−0.0465 (3)	0.11239 (9)	0.0360 (11)	
O2	0.0653 (10)	0.1296 (3)	0.09642 (10)	0.0441 (13)	
O3	0.3853 (10)	−0.1289 (3)	0.14414 (9)	0.0387 (12)	
O4	0.5930 (12)	0.2319 (4)	0.12531 (16)	0.0649 (17)	
H41	0.533 (17)	0.289 (4)	0.1312 (18)	0.078*	
H42	0.756 (6)	0.234 (7)	0.1210 (18)	0.078*	
Cl1	0.8942 (5)	−0.1606 (2)	0.01180 (5)	0.0729 (7)	
S1	0.1393 (3)	0.02651 (13)	0.10489 (3)	0.0315 (4)	

### Atomic displacement parameters (Å^2^)

**Table:**

	*U*^11^	*U*^22^	*U*^33^	*U*^12^	*U*^13^	*U*^23^
C1	0.031 (3)	0.027 (3)	0.027 (3)	−0.002 (3)	−0.003 (3)	0.006 (3)
C2	0.056 (5)	0.040 (4)	0.045 (4)	−0.006 (4)	0.008 (4)	−0.001 (4)
C3	0.059 (5)	0.051 (5)	0.043 (4)	−0.020 (4)	0.021 (4)	0.001 (4)
C4	0.043 (4)	0.049 (5)	0.035 (4)	−0.003 (4)	0.004 (3)	−0.008 (3)
C5	0.053 (5)	0.035 (4)	0.044 (4)	−0.001 (4)	0.005 (4)	−0.004 (3)
C6	0.042 (4)	0.037 (4)	0.031 (3)	−0.005 (4)	0.005 (3)	0.005 (3)
C7	0.030 (3)	0.037 (4)	0.030 (3)	0.002 (3)	0.005 (3)	0.002 (3)
C8	0.035 (4)	0.032 (4)	0.026 (3)	−0.003 (3)	0.004 (3)	−0.002 (3)
C9	0.040 (4)	0.032 (4)	0.028 (3)	0.000 (3)	0.004 (3)	−0.001 (3)
C10	0.036 (4)	0.056 (5)	0.029 (3)	−0.001 (4)	0.001 (3)	0.002 (4)
C11	0.045 (5)	0.087 (7)	0.033 (4)	−0.009 (5)	−0.011 (4)	−0.010 (4)
C12	0.072 (6)	0.058 (5)	0.048 (5)	−0.011 (5)	−0.004 (5)	−0.017 (4)
C13	0.055 (5)	0.038 (4)	0.038 (4)	−0.001 (4)	−0.005 (4)	−0.009 (3)
C14	0.045 (5)	0.088 (7)	0.055 (5)	0.005 (5)	−0.008 (4)	0.018 (5)
N1	0.029 (3)	0.028 (3)	0.033 (3)	−0.006 (3)	−0.005 (2)	−0.002 (2)
O1	0.032 (2)	0.039 (3)	0.037 (2)	−0.006 (2)	0.002 (2)	−0.001 (2)
O2	0.047 (3)	0.030 (3)	0.055 (3)	0.012 (2)	−0.009 (3)	0.004 (2)
O3	0.050 (3)	0.023 (3)	0.043 (3)	−0.004 (2)	−0.012 (2)	−0.001 (2)
O4	0.051 (4)	0.036 (3)	0.107 (5)	−0.003 (3)	−0.004 (4)	0.009 (3)
Cl1	0.0690 (15)	0.0909 (17)	0.0590 (12)	−0.0093 (14)	0.0315 (12)	−0.0208 (13)
S1	0.0309 (8)	0.0296 (8)	0.0338 (8)	0.0019 (8)	−0.0033 (7)	0.0031 (8)

### Geometric parameters (Å, °)

**Table:**

C1—C2	1.377 (9)	C9—H9	0.9300
C1—C6	1.392 (9)	C10—C11	1.369 (10)
C1—S1	1.747 (6)	C10—C14	1.505 (10)
C2—C3	1.384 (10)	C11—C12	1.380 (11)
C2—H2	0.9300	C11—H11	0.9300
C3—C4	1.371 (10)	C12—C13	1.392 (10)
C3—H3	0.9300	C12—H12	0.9300
C4—C5	1.357 (10)	C13—H13	0.9300
C4—Cl1	1.739 (7)	C14—H14A	0.9600
C5—C6	1.383 (9)	C14—H14B	0.9600
C5—H5	0.9300	C14—H14C	0.9600
C6—H6	0.9300	N1—S1	1.651 (5)
C7—O3	1.192 (7)	N1—H1N	0.86 (2)
C7—N1	1.400 (8)	O1—S1	1.426 (4)
C7—C8	1.520 (9)	O2—S1	1.432 (5)
C8—C13	1.380 (9)	O4—H41	0.84 (2)
C8—C9	1.398 (9)	O4—H42	0.84 (2)
C9—C10	1.387 (9)		
			
C2—C1—C6	120.5 (6)	C11—C10—C14	120.9 (7)
C2—C1—S1	120.0 (5)	C9—C10—C14	121.0 (7)
C6—C1—S1	119.5 (5)	C10—C11—C12	122.1 (7)
C1—C2—C3	118.9 (7)	C10—C11—H11	118.9
C1—C2—H2	120.6	C12—C11—H11	118.9
C3—C2—H2	120.6	C11—C12—C13	120.0 (7)
C4—C3—C2	120.1 (7)	C11—C12—H12	120.0
C4—C3—H3	119.9	C13—C12—H12	120.0
C2—C3—H3	119.9	C8—C13—C12	118.6 (7)
C5—C4—C3	121.5 (7)	C8—C13—H13	120.7
C5—C4—Cl1	119.0 (6)	C12—C13—H13	120.7
C3—C4—Cl1	119.4 (6)	C10—C14—H14A	109.5
C4—C5—C6	119.4 (7)	C10—C14—H14B	109.5
C4—C5—H5	120.3	H14A—C14—H14B	109.5
C6—C5—H5	120.3	C10—C14—H14C	109.5
C5—C6—C1	119.7 (6)	H14A—C14—H14C	109.5
C5—C6—H6	120.2	H14B—C14—H14C	109.5
C1—C6—H6	120.2	C7—N1—S1	122.9 (4)
O3—C7—N1	123.0 (6)	C7—N1—H1N	118 (4)
O3—C7—C8	123.7 (6)	S1—N1—H1N	114 (4)
N1—C7—C8	113.3 (6)	H41—O4—H42	113 (9)
C13—C8—C9	120.4 (6)	O1—S1—O2	119.5 (3)
C13—C8—C7	124.2 (6)	O1—S1—N1	108.8 (3)
C9—C8—C7	115.4 (6)	O2—S1—N1	104.8 (3)
C10—C9—C8	120.7 (6)	O1—S1—C1	109.8 (3)
C10—C9—H9	119.7	O2—S1—C1	107.9 (3)
C8—C9—H9	119.7	N1—S1—C1	105.2 (3)
C11—C10—C9	118.1 (7)		
			
C6—C1—C2—C3	−1.4 (11)	C9—C10—C11—C12	1.5 (11)
S1—C1—C2—C3	177.0 (6)	C14—C10—C11—C12	−179.5 (7)
C1—C2—C3—C4	0.5 (12)	C10—C11—C12—C13	−2.1 (12)
C2—C3—C4—C5	0.2 (13)	C9—C8—C13—C12	0.5 (11)
C2—C3—C4—Cl1	−178.5 (6)	C7—C8—C13—C12	178.7 (6)
C3—C4—C5—C6	0.0 (12)	C11—C12—C13—C8	1.0 (12)
Cl1—C4—C5—C6	178.6 (5)	O3—C7—N1—S1	−2.9 (9)
C4—C5—C6—C1	−0.8 (11)	C8—C7—N1—S1	177.3 (4)
C2—C1—C6—C5	1.5 (10)	C7—N1—S1—O1	46.8 (6)
S1—C1—C6—C5	−176.9 (5)	C7—N1—S1—O2	175.7 (5)
O3—C7—C8—C13	−159.8 (7)	C7—N1—S1—C1	−70.7 (5)
N1—C7—C8—C13	19.9 (9)	C2—C1—S1—O1	153.9 (5)
O3—C7—C8—C9	18.5 (9)	C6—C1—S1—O1	−27.6 (6)
N1—C7—C8—C9	−161.7 (5)	C2—C1—S1—O2	22.2 (6)
C13—C8—C9—C10	−1.0 (10)	C6—C1—S1—O2	−159.4 (5)
C7—C8—C9—C10	−179.4 (6)	C2—C1—S1—N1	−89.2 (6)
C8—C9—C10—C11	0.1 (10)	C6—C1—S1—N1	89.2 (6)
C8—C9—C10—C14	−179.0 (6)		

### Hydrogen-bond geometry (Å, °)

**Table:**

*D*—H···*A*	*D*—H	H···*A*	*D*···*A*	*D*—H···*A*
N1—H1N···O4	0.86 (2)	1.93 (2)	2.771 (8)	169 (6)
O4—H41···O1^i^	0.84 (2)	2.29 (7)	2.916 (7)	131 (8)
O4—H41···O3^i^	0.84 (2)	2.42 (6)	3.117 (8)	140 (8)
O4—H42···O2^ii^	0.84 (2)	2.35 (6)	3.022 (8)	137 (8)

Symmetry codes: (i) −*x*+1/2, *y*+1/2, *z*; (ii) *x*+1, *y*, *z*.

## References

[bb1] Gowda, B. T., Dou, S. Q. & Weiss, A. (1996). *Z. Naturforsch. Teil A*, **51**, 627–636.

[bb2] Gowda, B. T., Jyothi, K., Kozisek, J. & Fuess, H. (2003). *Z. Naturforsch. Teil A*, **58**, 656–660.

[bb3] Gowda, B. T., Svoboda, I. & Fuess, H. (2004). *Z. Naturforsch. Teil A*, **59**, 845–852.

[bb4] Jayalakshmi, K. L. & Gowda, B. T. (2004). *Z. Naturforsch. Teil A*, **59**, 491–500.

[bb5] Oxford Diffraction (2009). *CrysAlis CCD* and *CrysAlis RED* Oxford Diffraction Ltd, Yarnton, England.

[bb6] Sheldrick, G. M. (2008). *Acta Cryst.* A**64**, 112–122. 10.1107/S0108767307043930 18156677

[bb7] Spek, A. L. (2009). *Acta Cryst.* D**65**, 148–155. 10.1107/S090744490804362X PMC2631630 19171970

[bb8] Suchetan, P. A., Foro, S. & Gowda, B. T. (2011). *Acta Cryst.* E**67**, o22. 10.1107/S1600536810050087 PMC3050156 21522718

